# Paeoniflorin alleviates spinal cord injury in a cell model via regulating PTEN and PI3K/AKT signaling

**DOI:** 10.1515/biol-2025-1292

**Published:** 2026-02-24

**Authors:** Hongxiang Hong, Guanhua Xu, Jiajia Chen, Jinlong Zhang, Chunyan Ji, Zhiming Cui

**Affiliations:** Spine Surgery Department, Nantong First People's Hospital, Southeast University, Nantong, 226001, China; Department of Spine Surgery, The Second Affiliated Hospital of Nantong University, Nantong, 226001, China; Research Institute for Spine and Spinal Cord Disease of Nantong University, Nantong, 226001, China

**Keywords:** paeoniflorin, spinal cord injury, PTEN, PI3K/AKT signaling

## Abstract

Spinal cord injury (SCI) is a prevalent form of spinal cord dysfunction, and the discovery of new effective treatments remains a critical research focus. This study investigated the role of paeoniflorin in a lipopolysaccharide (LPS)-induced PC-12 cell model of SCI and examined the involvement of phosphatase and tensin homolog (PTEN) and the phosphoinositide 3-kinase/protein kinase B (PI3K/AKT) signaling pathway. Rat neuronal PC-12 cells were injured using LPS. Cell viability, proliferation, and apoptosis were assessed using the Cell Counting Kit-8, 5-ethynyl-2′-deoxyuridine (EdU), and Terminal deoxynucleotidyl transferase dUTP Nick End Labeling (TUNEL) assays, respectively. Western blotting and quantitative polymerase chain reaction were used to analyze PI3K, AKT, and PTEN expressions. PTEN was overexpressed to determine its functional role. LPS significantly reduced cell viability and proliferation, while increasing apoptosis. Paeoniflorin treatment ameliorated these injury markers in a dose-dependent manner, downregulated PTEN expression, and enhanced phosphorylated PI3K and AKT levels. PTEN overexpression counteracted the protective effects of paeoniflorin and its activation of PI3K/AKT signaling. Paeoniflorin alleviates LPS-induced cell injury in PC-12 cell model by inhibiting PTEN expression and subsequently activating the PI3K/AKT pathway.

## 1 Introduction

Spinal cord injury (SCI) is the most common spinal cord dysfunction. An average of 9.3 million new cases of traumatic SCI occur worldwide each year, and the global prevalence of SCI is estimated at 27.04 million [1], 2]. SCI is associated with high mortality and disability rates because disruption of neural connections between the spinal cord and central nervous system leads to irreversible outcomes, such as paraplegia, quadriplegia, and sensory dysfunction [3]. Currently, the treatment of SCI mainly includes surgery, drug therapy, and rehabilitation training; however, these methods have limited effects on improving long-term patient prognosis. Ultra-early decompression surgery after SCI has been found to effectively improve the recovery of motor and sensory functions of patients with SCI. Drug therapy includes the use of steroids such as methylprednisolone and dexamethasone. However, these drugs have a series of side effects; therefore, finding new and effective treatment strategies remains an essential focus in contemporary SCI research [4].

Traditional Chinese medicine can promote the rehabilitation of SCI by regulating qi and blood circulation, strengthening body resistance, eliminating pathogens, and improving microcirculation [5], 6]. Bushen Huoxue decoction is a commonly used Chinese medicine prescription, and its application in the recovery stage of SCI has achieved favorable outcomes. The study found that bromodeoxyuridine-positive cells and neuronal-nuclei-positive neurons increased significantly in the spinal cord treated with Bushen Huoxue decoction, confirming that neural stem cells differentiated into neurons [7]. *Paeonia lactiflora* Pall. is one of the components of Bushen Huoxue decoction, and its main active component, paeoniflorin, exhibits many pharmacological activities, including effects on spinal cord-related diseases. A study found that paeoniflorin can inhibit apoptosis of spinal cord tissue by inhibiting autophagy [8]. However, whether paeoniflorin can serve as a potential medication for the management of SCI remains unclear.

Phosphatase and Tensin homolog (PTEN) has been reported to participate in the process of different cellular events [9] and serves as a key regulator of the PI3K/AKT signaling [10]. In the nervous system, PTEN regulates neuronal growth, axonal regeneration, and synaptic plasticity. Studies have shown that inhibiting PTEN expression can promote the regeneration and functional recovery of neurons [11], 12]. However, whether paeoniflorin exerts its neuroprotective effects in SCI through modulation of the PTEN/PI3K/AKT axis remains unexplored.

Although SCI *in vivo* involves primary mechanical injury followed by a complex cascade of secondary injuries, *in vitro* models allow for focused research on specific pathological components. Neuroinflammation is the core driving factor of secondary injury, leading to neuronal apoptosis and loss of function. Numerous previous studies have confirmed that the lipopolysaccharide (LPS) – induced neuroinflammation model is a classic *in vitro* model for studying the mechanism of neuroprotective agents [13], 14]. The aim of this study is to investigate the potential protective effects of paeoniflorin in LPS induced spinal cord injury models of PC-12 cells, and to test the hypothesis that its mechanism involves PTEN downregulation and subsequent activation of the PI3K/AKT signaling pathway.

## 2 Materials and methods

### 2.1 Reagent source

PC-12 cells were obtained from the Cell Bank of China (Shanghai, China). Dulbecco’s modified Eagle’s medium (DMEM) was purchased from Gibco (USA), paeoniflorin was purchased from Chengdu Yuanshu Biotechnology Co., Ltd. (Sichuan, China), and LPS was purchased from Sigma-Aldrich (USA). The 5-ethynyl-2′-deoxyuridine (EdU) assay kit, Cell Counting Kit-8 (CCK-8), radioimmunoprecipitation assay (RIPA) lysate, bicinchoninic acid (BCA) protein quantitative kit, and western blot (WB) gel were acquired from Beyotime (Shanghai, China). Antibodies against PTEN, phosphorylated phosphoinositide 3-kinase (p-PI3K), PI3K, protein kinase B (AKT), phosphorylated AKT (p-AKT), and glyceraldehyde-3-phosphate dehydrogenase (GAPDH) were obtained from Abcam (USA). Polyvinylidene fluoride (PVDF) membranes were purchased from Millipore (USA), and the chemiluminescence imaging system was provided by Tanon (Shanghai, China).

### 2.2 Cell culture and grouping

PC-12 cells were cultured in DMEM with 10 % fetal bovine serum and 1 % penicillin/streptomycin at 37 °C and 5 % CO_2_. We used LPS to induce an inflammatory reaction and injury in PC-12 cells. We divided cells into the following groups (1): Control; (2) LPS (1 μg/mL) [13], 14]; (3) LPS + Paeoniflorin Low (10 μmol/L); (4) LPS + Paeoniflorin Medium (50 μmol/L); (5) LPS + Paeoniflorin High (100 μmol/L). The concentrations of paeoniflorin were selected based on previous studies demonstrating its bioactivity in neuronal models [8] and our pilot dose-response experiments.

### 2.3 Cell viability

Viability of PC-12 cells was evaluated using the CCK-8 assay. Briefly, PC-12 cells were placed in 96-well plates (5,000/well), and after treatment, CCK-8 (10 μL) was used to treat the cells for 2 h. Finally, absorbance was measured at 450 nm using a microplate reader.

### 2.4 5-ethynyl-2′-deoxyuridine assay

After grouping, PC-12 cells were incubated with EdU (10 μM) for 2 h. The cells were subsequently fixed and permeabilized, followed by the click reaction and DAPI staining. Stained cells were imaged, and the number of EdU^+^ cells was quantified.

### 2.5 TUNEL staining

After grouping, PC-12 cells were fixed and permeabilized, and the Terminal deoxynucleotidyl transferase dUTP Nick-End Labeling (TUNEL) assay was performed following the manufacturer’s instructions. TUNEL^+^ cells were observed and counted using a fluorescence microscope.

### 2.6 Cell transfection

PC-12 cells were inoculated into 6-well plates and cultured until reaching confluence. PTEN overexpression (PTEN-OE) plasmids or empty vectors were transfected using Lipofectamine 3,000 (Thermo Fisher Scientific, USA). After 48 h, the cells were harvested for follow-up experiments.

### 2.7 Reverse transcription quantitative polymerase chain reaction

Total ribonucleic acid (RNA) was isolated from PC-12 cells subjected to different treatments using TRIzol reagent. The RNA samples were then reverse-transcribed into complementary DNA. Quantitative polymerase chain reaction (PCR) was performed using the Easy-Load™ PCR Master Mix (Beyotime) on the ABI 7500 Real-time PCR system (Applied Biosystems). The PCR conditions were as follows: initial denaturation at 95 °C for 10 min, followed by 40 cycles of 95 °C for 15 s and 60 °C for 1 min. Each sample was set up in three wells, and GAPDH was used for standardization. PTEN mRNA expression was calculated using the 2-ΔΔCT method.

### 2.8 Western blot

After cell collection, cell samples were lysed in RIPA buffer on ice for 30 min. Following pyrolysis, the lysates were centrifuged at 12,000 rpm and 4 °C for 15 min, and protein concentrations of each sample were determined using the BCA method (Pierce, Thermo Fisher Scientific). Proteins were separated by sodium dodecyl sulphate-polyacrylamide gel electrophoresis and transferred onto PVDF membranes. After the transfer, membranes were blocked with 5 % skim milk at room temperature for 1 h to prevent nonspecific antibody binding. Subsequently, membranes were incubated overnight with primary antibodies against PTEN (1:1000), p-PI3K (1:1000), PI3K (1:1000), AKT (1:1000), p-AKT (1:1000), and GAPDH (1:1000). On Day 2, after washing, membranes were incubated with horseradish peroxidase-conjugated secondary antibodies (1:5000) for 1 h at room temperature. Proteins (20–30 μg per lane) were loaded for electrophoresis. After incubation, protein bands were visualized using an enhanced chemiluminescence reagent, and images were captured with a chemiluminescence imaging system.

### 2.9 Statistics

Statistical analyses were performed using SPSS 22.0. Data are presented as mean ± standard deviation (SD) from at least three independent experiments (*n* ≥ 3). Comparisons among multiple groups were performed using one-way analysis of variance (ANOVA) followed by Tukey’s post-hoc test. Statistical significance was defined as *p* < 0.05.

## 3 Results

### 3.1 Paeoniflorin promoted LPS-treated PC-12 cell proliferation and inhibited apoptosis

**Figure 1:**
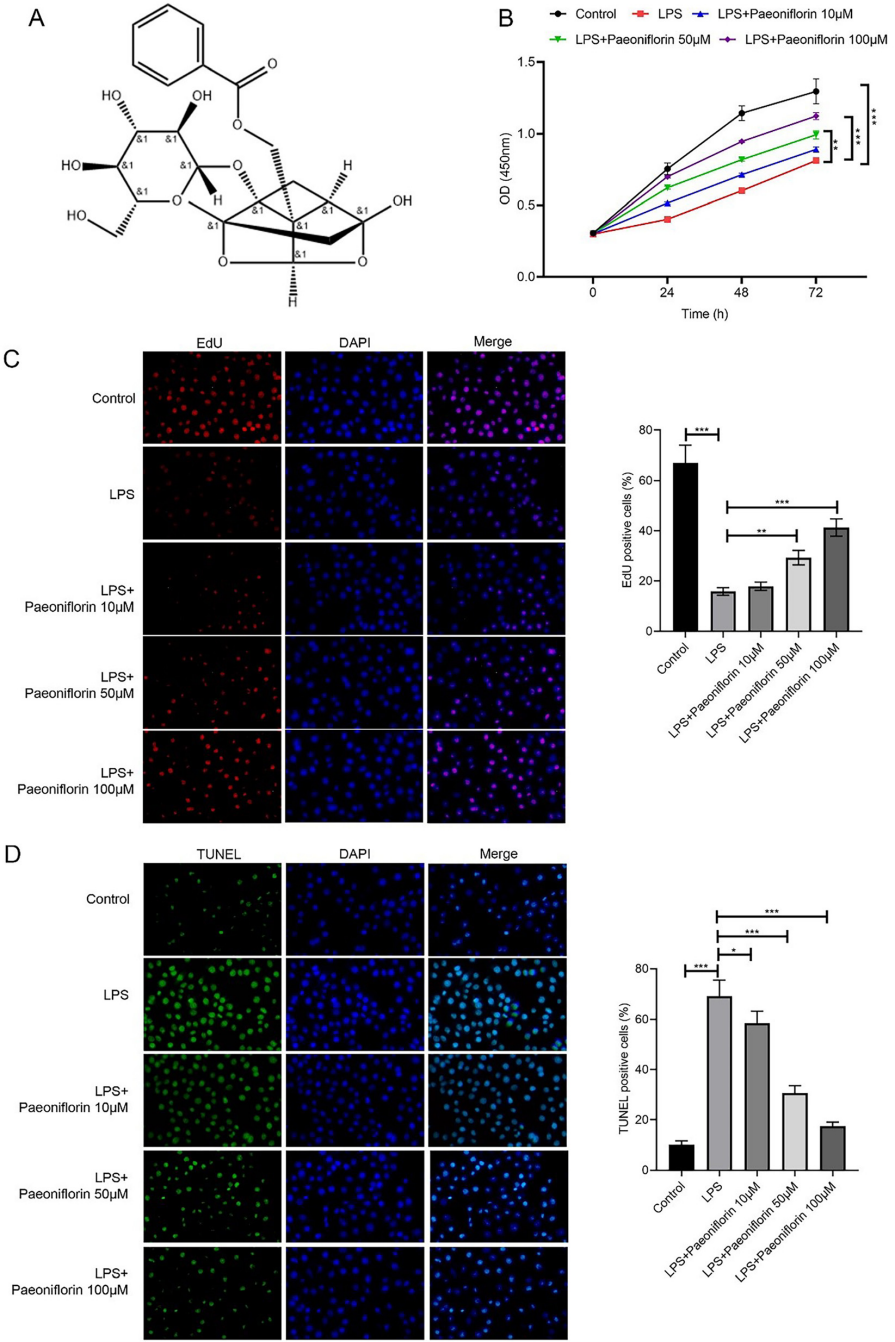
Effects of different doses of paeoniflorin on proliferation and apoptosis of LPS-treated PC12 cells. (A) Chemical structure of paeoniflorin. (B) Cell viability of LPS-treated PC-12 cells following treatment with varying concentrations of paeoniflorin, assessed by CCK-8 assay. (C) Representative images of EdU staining (red, for proliferation) and DAPI staining (blue, for nuclei) of PC-12 cells under different treatment conditions. The right panel quantifies the percentage of EdU-positive cells. (D) Representative images of TUNEL staining (green, for apoptosis) and DAPI staining (blue, for nuclei) of PC-12 cells under different treatment conditions. The right panel quantifies the percentage of TUNEL-positive cells. Data are presented as mean ± SD (*n* ≥ 3). Significance is indicated as *p < 0.05, **p < 0.01, ***p < 0.001, versus LPS group.

Figure 1A shows the chemical composition of paeoniflorin. To evaluate the effects of paeoniflorin on LPS-treated PC-12 cells, CCK-8, EdU, and TUNEL assays were performed. LPS significantly reduced cell viability and proliferation (Figure 1B and C, *p* < 0.001), while increasing apoptosis (Figure 1D, *p* < 0.001). Paeoniflorin treatment dose-dependently restored cell viability and proliferation, and attenuated apoptosis, with 100 µM showing the most pronounced effects (Figure 1B–D, *p* < 0.001). This concentration was therefore selected for subsequent experiments.

### 3.2 Paeoniflorin restores LPS-impaired PI3K/AKT signaling in a dose-dependent manner

**Figure 2:**
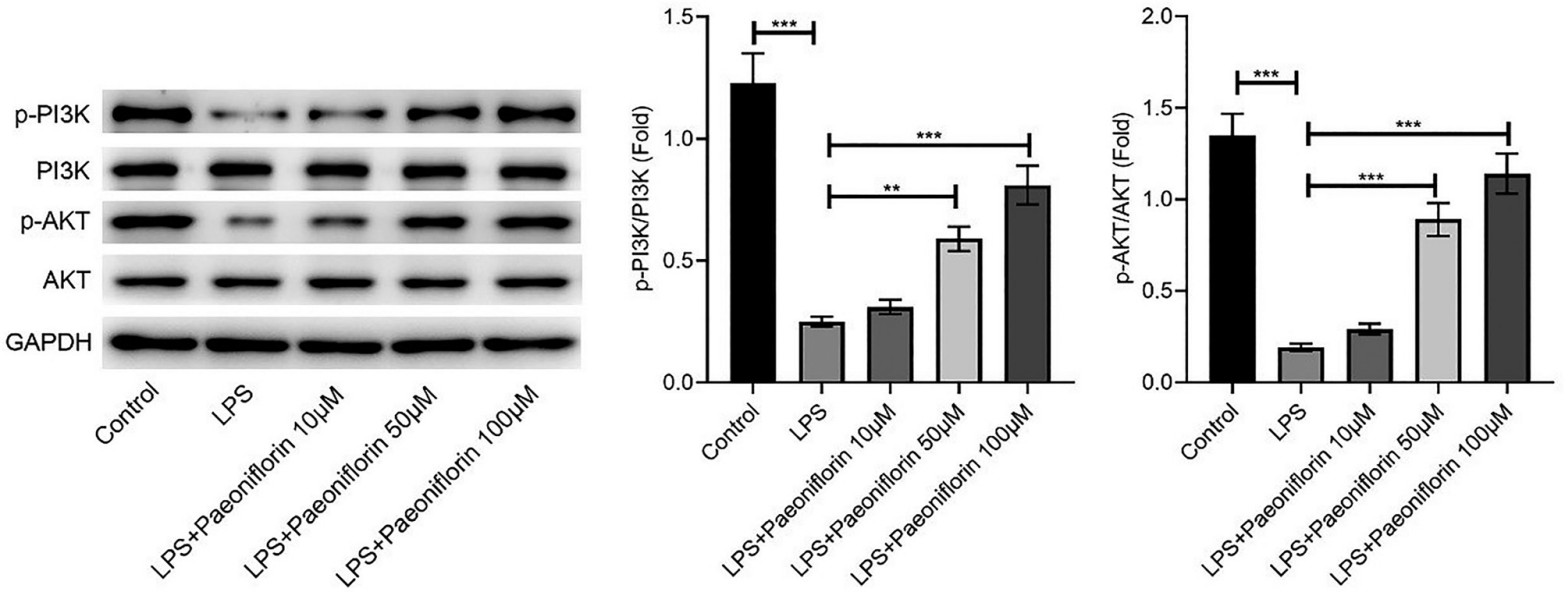
Paeoniflorin enhances PI3K/AKT phosphorylation in LPS-treated PC-12 cells. Western blot analysis of p-PI3K, PI3K, p-AKT, AKT, and GAPDH protein expression in PC-12 cells treated with control, LPS, or LPS + varying concentrations of paeoniflorin (10, 50, 100 μM). GAPDH was used as a loading control. Representative blots are shown. Quantification of p-PI3K/PI3K and p-AKT/AKT ratios are presented as mean ± SD (*n* ≥ 3). Significance is indicated as **p < 0.01, ***p < 0.001, versus LPS group.

Western blot analysis revealed that LPS significantly inhibited the PI3K/AKT pathway, evidenced by decreased ratios of p-PI3K/PI3K and p-AKT/AKT (Figure 2, *p* < 0.001). Paeoniflorin treatment (50 and 100 μM) effectively reversed this inhibition, significantly restoring the phosphorylation levels of both PI3K and AKT (*p* < 0.01 and *p* < 0.001, respectively).

### 3.3 Paeoniflorin regulates PTEN expression in LPS-treated PC-12 cells

**Figure 3:**
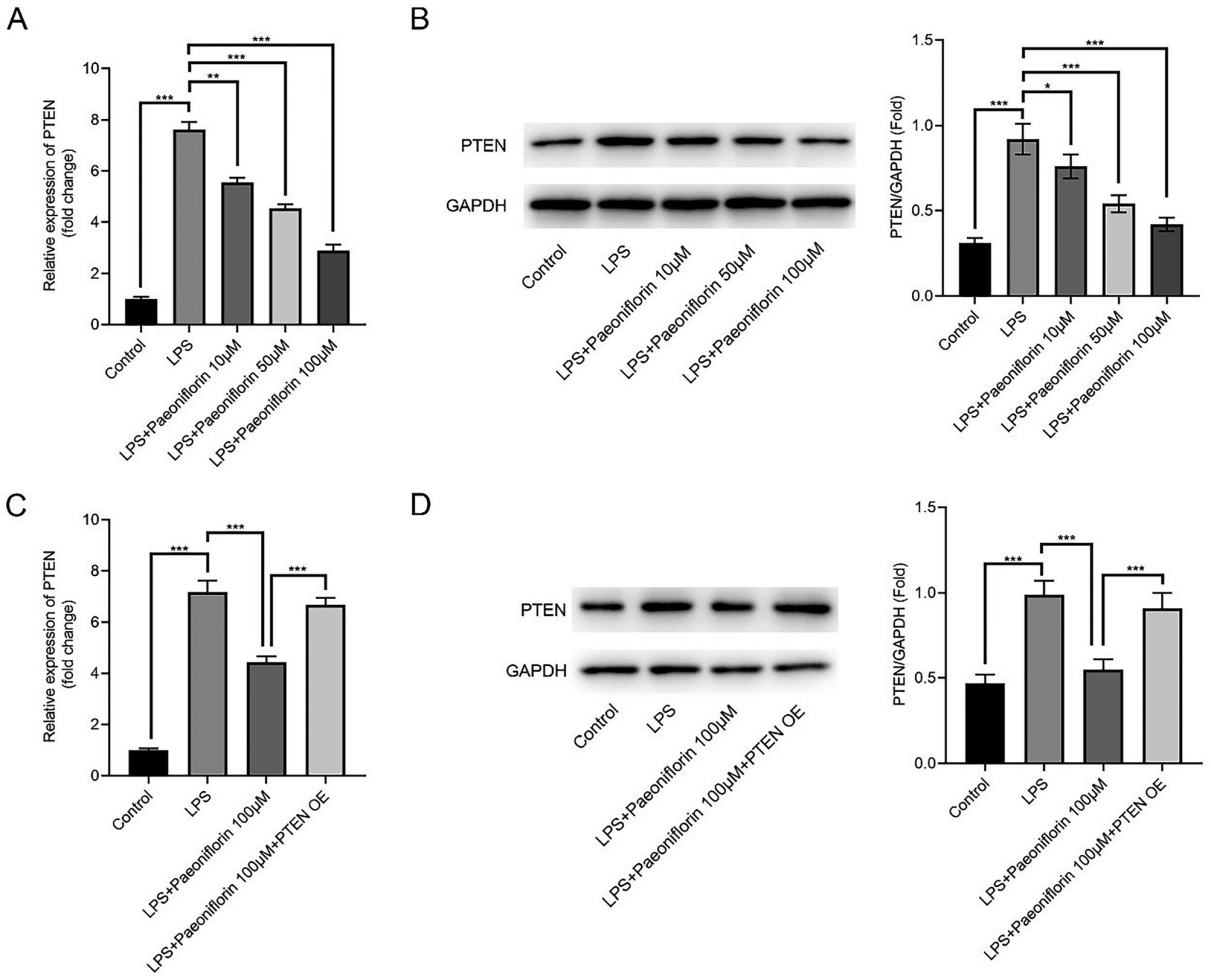
Paeoniflorin regulates PTEN expression in LPS-treated PC-12 cells. (A) PTEN mRNA expression in LPS-treated PC-12 cells after treatment with varying concentrations of paeoniflorin, determined by RT-qPCR. (B) Representative Western blot images and densitometric quantification of PTEN protein expression in LPS-treated PC-12 cells after treatment with varying concentrations of paeoniflorin. GAPDH was used as a loading control. (C) PTEN mRNA expression in LPS-treated PC-12 cells after 100 μM paeoniflorin treatment with or without PTEN overexpression (PTEN-OE), determined by RT-qPCR. (D) Representative Western blot images and densitometric quantification of PTEN protein expression in LPS-treated PC-12 cells after 100 μM paeoniflorin treatment with or without PTEN overexpression (PTEN-OE). GAPDH was used as a loading control. Data are presented as mean ± SD (*n* ≥ 3). Significance is indicated as *p < 0.05, ***p < 0.001 versus LPS group or versus LPS + Paeoniflorin (100 μM) group as indicated by the brackets.

LPS significantly increased PTEN mRNA and protein expression in PC-12 cells (*p* < 0.001). Paeoniflorin dose-dependently reversed this upregulation (Figure 3A and B, *p* < 0.01 and *p* < 0.001, respectively). Furthermore, overexpression of PTEN (PTEN-OE) counteracted the inhibitory effect of 100 μM paeoniflorin on PTEN levels in LPS-treated cells (Figure 3C and D, *p* < 0.001), suggesting that paeoniflorin acts, at least in part, by downregulating PTEN.

### 3.4 Overexpression of PTEN alleviates the protective effects of paeoniflorin on LPS-treated PC-12 cells

**Figure 4:**
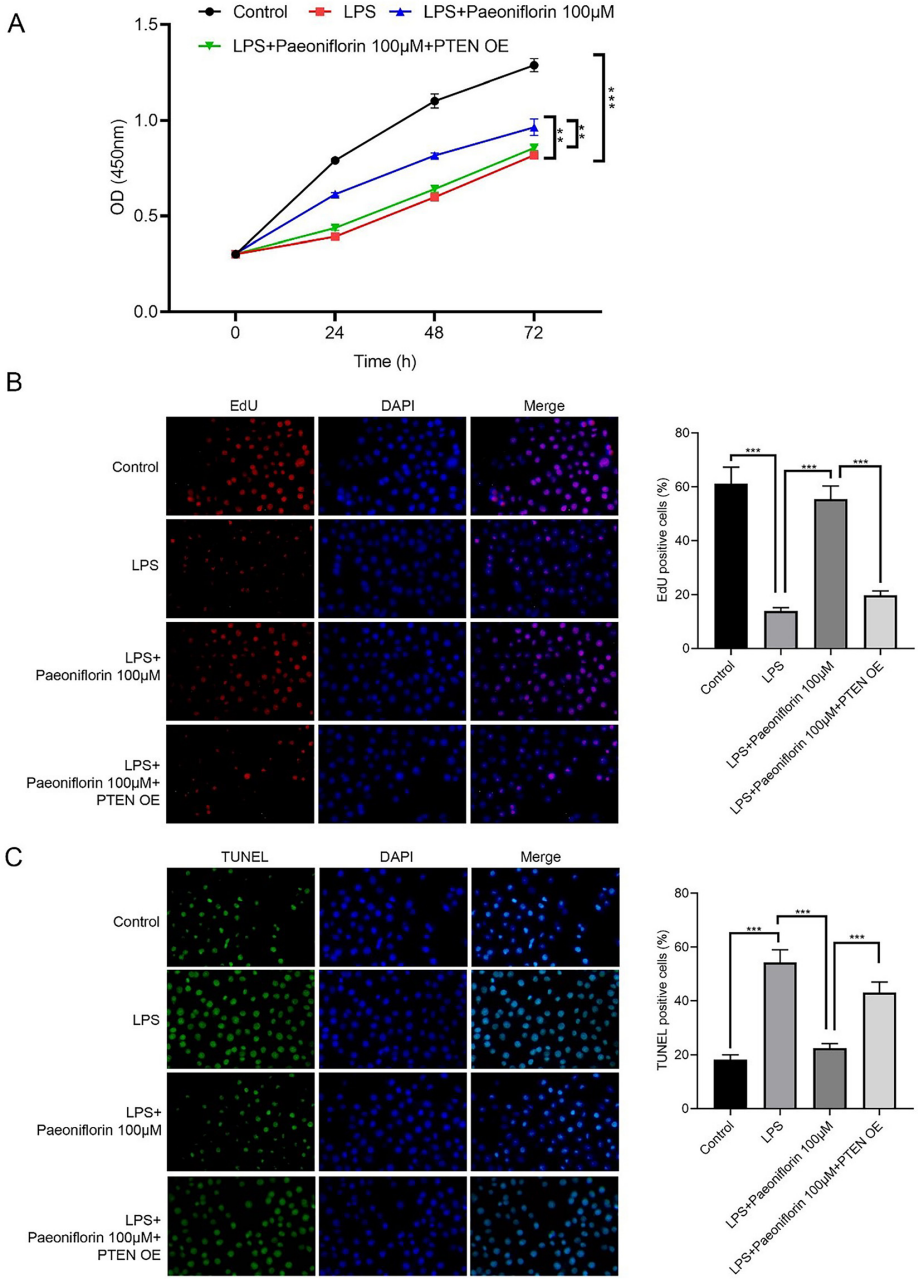
Effects of paeoniflorin + PTEN overexpression on LPS-treated PC12 cells. (A) Cell viability of LPS-treated PC-12 cells following treatment with varying concentrations of paeoniflorin, assessed by CCK-8 assay. (B) Representative images of EdU staining (red, for proliferation) and DAPI staining (blue, for nuclei) of PC-12 cells under different treatment conditions. The right panel quantifies the percentage of EdU-positive cells. (C) Representative images of TUNEL staining (green, for apoptosis) and DAPI staining (blue, for nuclei) of PC-12 cells under different treatment conditions. The right panel quantifies the percentage of TUNEL-positive cells. Data are presented as mean ± SD (*n* ≥ 3). Significance is indicated as **p < 0.01, ***p < 0.001, versus LPS group or versus LPS + Paeoniflorin (100 μM) group as indicated by the brackets.

Figure 4 shows that PTEN-OE attenuates the protective effect of paeoniflorin. Viability of PC-12 cells in the LPS + paeoniflorin + PTEN-OE group was significantly lower than that in the LPS + paeoniflorin group (Figure 4A, *p* < 0.01). EdU assay indicated that the proliferation of PC-12 cells in the LPS + paeoniflorin + PTEN-OE group was markedly lower than that in the LPS + paeoniflorin group (Figure 4B, *p* < 0.001). TUNEL staining further revealed increased apoptosis of PC-12 cells in the LPS + paeoniflorin + PTEN-OE group compared with the LPS + paeoniflorin group (Figure 4C, *p* < 0.001).

### 3.5 Paeoniflorin regulates LPS-induced PC-12 cell injury by inhibiting PTEN through affecting P13K/AKT signaling

**Figure 5:**
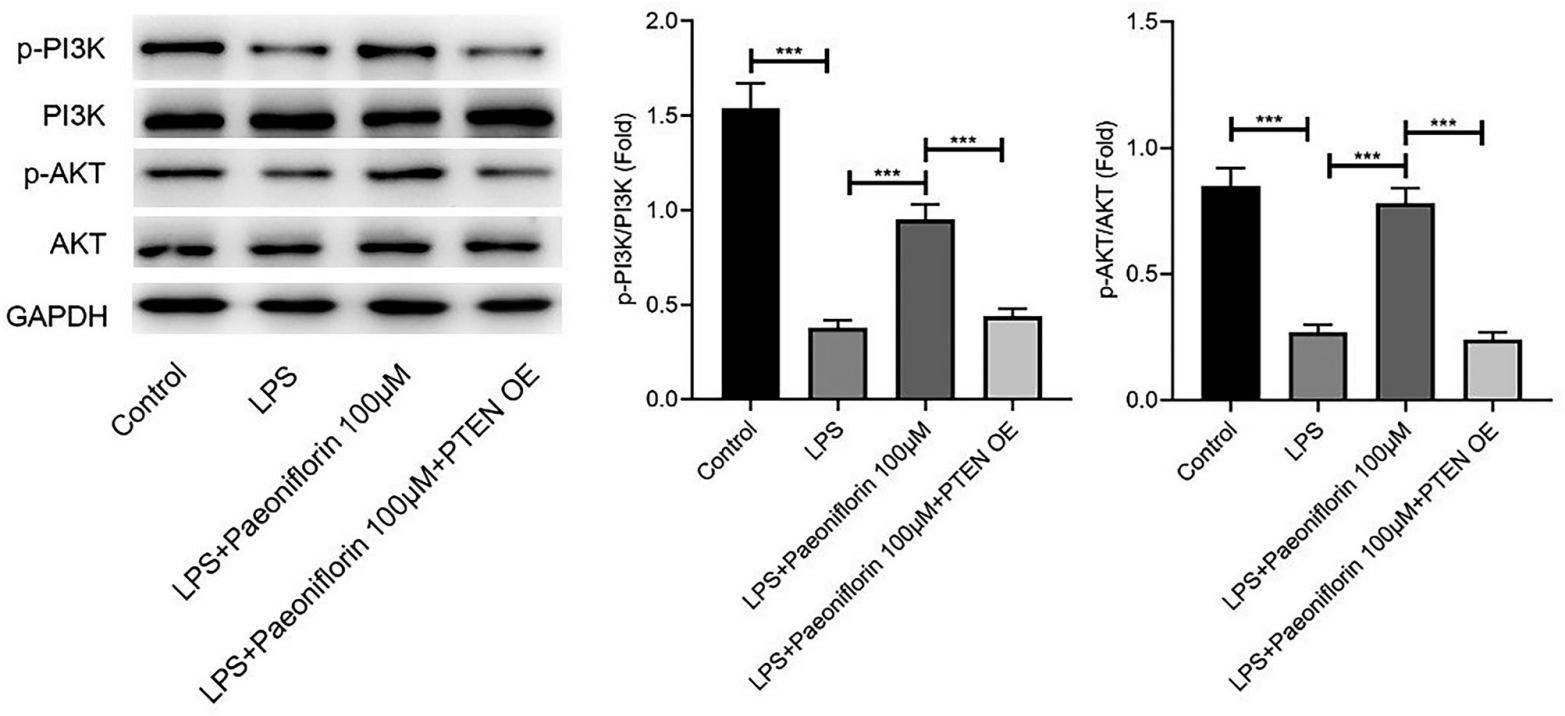
Paeoniflorin regulates LPS-induced PC-12 cell injury by inhibiting PTEN through affecting P13K/AKT signaling. Representative Western blot images showing the protein expression levels of p-PI3K, PI3K, p-AKT, AKT, and GAPDH in PC-12 cells treated with Control, LPS, LPS + Paeoniflorin (100 μM), and LPS + Paeoniflorin (100 μM) + PTEN-OE. GAPDH was used as a loading control. Representative blots are shown. Quantification of p-PI3K/PI3K and p-AKT/AKT ratios are presented as mean ± SD (*n* ≥ 3). Significance is indicated as ***p < 0.001, versus LPS group or versus LPS + Paeoniflorin (100 μM) group as indicated by the brackets.

WB analysis showed that the levels of p-PI3K and p-AKT were significantly increased in the LPS + paeoniflorin group compared with that in the LPS group (Figure 5, *p* < 0.001). In contrast, PTEN-OE reversed the effects of paeoniflorin on LPS-induced PC-12 cells, thereby reducing p-PI3K and p-AKT expressions (Figure 5, *p* < 0.001).

## 4 Discussion

This study demonstrated that paeoniflorin alleviates LPS-induced PC-12 cell injury, and the possible mechanism may involve PTEN inhibition and suppression of the PI3K/AKT signaling pathway.

SCI is a serious disease of the central nervous system that leads to permanent neurological impairment below the site of damage, placing a heavy burden on both patients and society. Its pathological processes include primary and secondary injuries, encompassing inflammatory responses, autophagy, blood-brain barrier destruction, and neuronal apoptosis [15]. Due to the limited regenerative capacity of neurons, repair after SCI is challenging, often resulting in irreversible damage. LPS can induce inflammatory responses and subsequent cell damage. In the nervous system, LPS triggers neuroinflammation and promotes neuronal apoptosis; therefore, it has been used in previous studies to establish SCI cell models [16], 17]. In this study, an SCI cell model was created by treating PC-12 cells with LPS to simulate a neuroinflammatory environment. Paeoniflorin, the main active component of *P. lactiflora*, exhibits a wide range of pharmacological activities, including restoration of mitochondrial function and inhibition of neuroinflammation, oxidative stress, and apoptosis in nervous system disorders [18], 19]. Previous studies have shown that Paeoniflorin can improve symptoms of various neurological diseases [20], [21], [22]. In our study, paeoniflorin promoted proliferation and inhibited apoptosis in LPS-treated PC12 cells, thereby confirming its neuroprotective effect.

PTEN is a critical negative regulator that dephosphorylates the phospholipid product PI (3,4,5) P3 of PI3K, thus inhibiting PI3K/AKT signaling [11]. In this study, we found that LPS increased PTEN expression in PC-12 cells, whereas paeoniflorin effectively reduced PTEN expression. These findings are consistent with previous studies, indicating that PTEN expression is upregulated after nerve injury [23]. Additionally, PI3K/AKT signaling has been reported to prevent nerve injury and neuroinflammation caused by ischemic stroke [24], and targeting PTEN may modulate PI3K/AKT signaling while improving apoptosis induced by brain ischemia/reperfusion injury [25]. In this study, we observed a decrease in PTEN expression after treatment with paeoniflorin, accompanied by changes in PI3K and AKT phosphorylation levels. Importantly, we found that overexpression of PTEN can partially counteract the protective effect of paeoniflorin on cell viability, suggesting that PTEN may be involved in the mechanism of paeoniflorin action. These *in vitro* experimental results provide preliminary mechanistic clues for understanding the cell protective effect of paeoniflorin.

This study has several limitations. Only one *in vitro* cell model was studied, and *in vivo* verification is lacking. Furthermore, this study focused solely on the effects of paeoniflorin on LPS-treated PC-12 cells, without examining its protective effects on other nerve cells. Therefore, future research is needed to validate the neuroprotective roles of paeoniflorin and to investigate its protective effects on different types of nerve cells.

However, this study has limitations that should be acknowledged. First, although the LPS-induced PC-12 cell model is widely used to study neuroinflammation and neuroprotection, it is a simplified *in vitro* system. The PC-12 cell line, while a valuable neuronal-like model, does not fully recapitulate the complex cellular diversity of the spinal cord, which includes primary neurons, astrocytes, microglia, and oligodendrocytes. The lack of validation using more physiologically relevant primary cells or co-culture systems that better mimic the multicellular interactions within the injured spinal cord microenvironment limits the direct extrapolation of our findings. Second, the LPS model primarily mimics the inflammatory component of secondary SCI, but does not incorporate the mechanical trauma that initiates primary injury. Consequently, our findings represent an initial exploration of paeoniflorin’s effect on a specific aspect of SCI pathology. The translational relevance of the observed protective effects and the proposed PTEN/PI3K/AKT mechanism require robust validation in established animal models of contusion or compression SCI. Future studies should employ such *in vivo* models to assess functional recovery and explore the effects of paeoniflorin across the diverse cell populations involved in SCI pathophysiology.

This study revealed that paeoniflorin exerts neuroprotective effects in spinal cord injury by regulating the PTEN/PI3K/AKT pathway. This discovery, along with multiple recent studies, emphasizes the importance of multi-target intervention in the treatment of SCI. Future research can further expand the mechanism of action and therapeutic potential of paeoniflorin from multiple dimensions based on this. Firstly, considering the key role of the SIK2/AIM2 signaling axis in regulating mitochondrial dynamics and neuroinflammation mediated by microglia [26], as well as the mechanism of betulinic acid regulating autophagy and mitochondrial autophagy through the AMPK mTOR TFEB pathway and inhibiting cell pyroptosis [27], the study should explore whether paeoniflorin is also involved in regulating the mitochondrial quality control system, including its regulatory effect on autophagy/mitochondrial autophagy pathways, its impact on ROS homeostasis, and whether it affects inflammasome activity and cell pyroptosis processes such as AIM2 by intervening in Drp1 dependent mitochondrial division and mtDNA leakage. Secondly, considering the important role of the brain gut axis in neural repair and the mechanism by which gut microbiota such as *Akkermansia muciniphila* affects neuroimmune homeostasis by regulating inflammatory factors, neurotransmitters, and short chain fatty acids [28], 29], it is necessary to explore whether the systemic anti-inflammatory and neuroprotective effects of paeoniflorin involve the regulation of gut microbiota and its potential regulatory effects on brain gut axis signals. For example, changes in gut microbiota composition, gut barrier function, and related metabolites in serum/brain tissue after paeoniflorin intervention can be verified. At the same time, lysosomal membrane permeability (LMP) triggers inflammatory cascade reactions and various forms of programmed cell death in central nervous system damage through the release of proteases and other contents, making the study of whether paeoniflorin exerts neuroprotective effects by stabilizing lysosomal membrane structure and inhibiting LMP a highly promising research direction [30]. Finally, based on the above directions, the long-term efficacy of paeoniflorin on neurological function recovery should be systematically evaluated in animal models, and its synergistic effects with existing treatment strategies (such as regulating mitochondrial function or regulating gut microbiota) should be explored to provide more comprehensive experimental evidence for its clinical application.

In conclusion, this study provides evidence that paeoniflorin protects against neuronal injury and suppresses microglial inflammation through regulation of the PTEN/PI3K/AKT pathway. These findings highlight the multi-faceted therapeutic potential of paeoniflorin in SCI management and provide a solid foundation for future preclinical and clinical investigations.
